# A Plea for Moderation

**Published:** 1895-09

**Authors:** B. Holly Smith

**Affiliations:** Baltimore, Md.


### A PLEA FOR MODERATION.[1]

BY DR. B. HOLLY SMITH, BALTIMORE, MD.

IT is becoming a matter for serious consideration and thought how far the work of preliminary training shall extend, and what portion of a man's life shall be given to preparation pure and simple.

Among educators this question has been most thoughtfully and ably discussed, and there are not lacking adherents of the practical side of it who would limit the time spent in getting ready for the

---

[1] Read before the Odontological Society of Pennsylvania, March 9, 1895.

work of life. Theoretically, preparation should be perfect, and, theoretically, no time is misspent which is devoted to thorough, elaborate, and consistent training.

We have been educated up to the point of expecting higher things, and the cry all along the line has been "elevate the standard and raise the dignity of the profession." This has been true, nay, is true, not only in professional branches of education, but in all departments of preliminary work.

Secondary schools all over the country are presenting curricula whose faithful performance calls for the extended and elaborate work of a university. Colleges think it beneath their dignity to confine their efforts to the former contracted limits, and within the trackless maze of the university proper all is lost sight of to the ordinary mortal beneath the cabalistic and mysterious terms of individual "investigation and original work."

The laboratory, the library, the lecture-room, the pen, the printing-press feed and fan the flame, and promote the activity of these swarming original workers. Many are the manuscripts; monographs are multiplied; bloated volumes, immature digests, and accumulations of valueless trifles, the results of frantic strife for doctors' degrees, load the overcrowded shelves of groaning libraries, until we remember with some satisfaction that "of making many books there is no end, and much study is a weariness to the flesh," and would be glad "to hear the conclusion of the whole matter." The fact is, the world has begun to differentiate, nay, has progressed so far along in the way of differentiation that it is extremely difficult to say where the thing will cease. Specializing is the fascinating pursuit which claims the best efforts and attention of our ardent young workers, and getting ready to specialize is the subject that engrosses the stern, restrictive, disciplinary watchfulness of our vigilant leaders and thinkers. What is necessary? What degree of training? What amount of time? How much study? If the limit for these requirements is too greatly contracted, an injury is done the profession and the public. Our conservators, our jealous guardians of professional interests are ever on the alert to guard against this, and elevation of the standard of professional dental education has been the constant aim and care of every devoted lover of dentistry. Thanks to the noble and united efforts of devoted men this end has been most successfully attained. All honor to their efforts. All praise for their success. But are we prepared to go to extremes in this matter? Is there not a golden mean, whose practical and certain attainment is of more real value than a theoretical elevation of standard which will discourage and prohibit or else beget dangerous practices of evasion and circumvention? To most questions there is what may be called the utilitarian side, and it is certainly true of this particular one.

What is practicable, what is attainable, is better than the ideal and visionary. The former can inspire hope and promise of success, the latter fills us with the gloom of discouragement or renders us guilty of the duplicity of self-deceit.

The course of preparatory dental study has been lengthened to three years, and preliminary examinations are required in accordance with the recommendations and suggestions of the Association of Faculties. This great improvement is meeting with unquestioned approval everywhere, and the benefits conferred upon the profession are not to be doubted for one moment.

Why not go further? say some of our enthusiasts. Why not extend the course to four years, and increase the requirements of the preliminary examination? Can we have too much of a good thing?

Undoubtedly the matter under discussion is simply a question of degree, and it becomes our duty as fair-minded, temperate, impartial men to decide whether we are prepared to side with the extremists on this question.

Is it not the more reasonable plan? Is it not the better and more advisable policy to adjust ourselves to the acknowledged excellent conditions now existing before we create those of a more difficult and exacting nature? Let us make haste slowly in this matter. If we do well in practising the task we have set for ourselves in theory, I think we may well rejoice in the attainment of a great and visible good,—something not to be exchanged for the imperfect realization of more vigorous requirements.

We all know that as far as successful and creditable performance of the real work of our profession goes, we have made ample provision for preparation both in point of time and of requirements.

The practical and successful members of our profession would probably gaze askance at a summons to produce a good knowledge of the classics as an evidence of the capacity to treat diseased teeth. How would the majority of us feel if called upon before giving a clinic to construe ten lines from Virgil, or to scan an ode of Horace. And yet these tests are proposed. A knowledge of the classics is a most desirable thing, so of mathematics,—refining, elevating, cultivating,—but had we not better leave such matters to schools whose special function it is to teach them; and are we prepared to say to a candidate for admission into the profession of dentistry, "You don't know Latin; then you are not fit to study dentistry."

It seems to me we must limit preliminary requirements both as to subjects and amount; plainly to such subjects alone as are needed for the practical work in lecture-room and office. We have nothing to do with those ornaments and embellishments which are the flowers of culture. We do not dispute that the moral effect would be greater if every dentist were also a savant, —but would the real pain-alleviating power of the profession be increased?

As dentists, I take it, the degree of a man's ornamental culture does not concern us, but the quality and quantity of his necessary scientific and professional knowledge, and that only so far as it is a protection and a guarantee to the profession against ignorance and quackery.

We may resolve that it is a most creditable thing to know Latin, but can we legislate that one cannot be a dentist unless he does know Latin?

Now as to lengthening the time of study in dental colleges. I simply think we are not at present prepared to do it.

*Reasons.*—1. The dental profession through its institutions of education is already fully abreast of the other professions in its requirements.

2. There is no necessity of overlooking the educational influence of the dental society and the dental journal, nor of expecting that every student who emerges from a dental school should have a finished education; he is only to be equipped with the knowledge which will enable him to grapple with the problem of his professional life; and there will still be much that can be acquired in no other way than by contact with his brethren of the dental profession and in the actual handling of a practice. If we carefully fit good men to profit by these larger opportunites, have we not fulfilled our mission as teachers?

3. We have no right to refuse to recognize the opportunity to establish post-graduate schools, whose influence would benefit in broadening and deepening the culture of the few who could afford these privileges. All schools and all students should not be required to go so far; it would work an injustice and cause the arrest of many a career.

4. We cannot afford to limit the number of those entering our profession to such as are sufficiently well off in this world's goods to give so extended a time to preliminary training; they are too few.

5. Experience teaches that a man too long kept in leading-strings—that is, under the guidance and direction of teachers beyond the period of greatest enthusiasm and activity—is forever handicapped.

The difference in the two methods may be readily seen by a comparison between the practical efficiency of our own dentists and those of Germany, where preliminary training is carried to its ultimate conclusion.

But beyond and above all am I unalterably opposed to any measures designed to prohibit young and reputable men from entering upon the study of this profession simply as a protection to ourselves.

Such a course would be a crying injustice to them and of suicidal folly to us. The theorists who advocate it have not considered.

Do we deem our profession worthy, let us invite talent to its ranks and stimulate the deserving to join us. No selfish policy can protect us,—and from such protection we should all pray for deliverance.

What do we fear? What do we hope? Our brief span must soon run out, and to whose care must we confide the glorious enterprise of our lives except to the noble youth who glows with the enthusiasm of a generous and honorable emulation.

